# Computer-Aided Strategy on 5-(Substituted benzylidene) Thiazolidine-2,4-Diones to Develop New and Potent PTP1B Inhibitors: QSAR Modeling, Molecular Docking, Molecular Dynamics, PASS Predictions, and DFT Investigations

**DOI:** 10.3390/molecules29040822

**Published:** 2024-02-10

**Authors:** Nour-El Houda Derki, Aicha Kerassa, Salah Belaidi, Maroua Derki, Imane Yamari, Abdelouahid Samadi, Samir Chtita

**Affiliations:** 1 VTRS Laboratory, Faculty of Sciences, University of El Oued, P.O. Box 789, El Oued 39000, Algeriaaichachimie1@gmail.com (A.K.);; 2 Group of Computational and Medicinal Chemistry, Laboratory of Molecular Chemistry and Environment, University of Biskra, P.O. Box 145, Biskra 07000, Algeria; prof.belaidi@gmail.com; 3 Laboratory of Analytical and Molecular Chemistry, Faculty of Sciences Ben M’Sik, Hassan II University of Casablanca, Sidi Othman, Casablanca P.O. Box 7955, Morocco; 4 Department of Chemistry, College of Science, UAEU, Al Ain P.O. Box 15551, United Arab Emirates

**Keywords:** thiazolidine-2,4-diones, PTP1B inhibitors, QSAR, docking, molecular dynamics, ADMET, PASS predication, DFT

## Abstract

A set of 5-(substituted benzylidene) thiazolidine-2,4-dione derivatives was explored to study the main structural requirement for the design of protein tyrosine phosphatase 1B (PTP1B) inhibitors. Utilizing multiple linear regression (MLR) analysis, we constructed a robust quantitative structure–activity relationship (QSAR) model to predict inhibitory activity, resulting in a noteworthy correlation coefficient (R^2^) of 0.942. Rigorous cross-validation using the leave-one-out (LOO) technique and statistical parameter calculations affirmed the model’s reliability, with the QSAR analysis revealing 10 distinct structural patterns influencing PTP1B inhibitory activity. Compound **7e(ref)** emerged as the optimal scaffold for drug design. Seven new PTP1B inhibitors were designed based on the QSAR model, followed by molecular docking studies to predict interactions and identify structural features. Pharmacokinetics properties were assessed through drug-likeness and ADMET studies. After that density functional theory (DFT) was conducted to assess the stability and reactivity of potential diabetes mellitus drug candidates. The subsequent dynamic simulation phase provided additional insights into stability and interactions dynamics of the top-ranked compound **11c**. This comprehensive approach enhances our understanding of potential drug candidates for treating diabetes mellitus.

## 1. Introduction

Diabetes mellitus (DM) is a metabolic disorder that poses a significant threat to life and is characterized by elevated levels of glucose in the bloodstream. The global incidence of diabetes is rapidly increasing and has become a major cause of concern. The International Diabetes Federation reports that around 537 million adults are presently afflicted with the condition, and this number is projected to rise to 643 million by 2030 [1,2]. There are two main sub-types of diabetes mellitus (DM) that are commonly recognized. Type 1 diabetes is an autoimmune condition that affects pancreatic cells, reducing or impairing insulin production, whereas type 2 diabetes is caused by impairment of pancreatic beta cells, limiting the individual’s capacity to utilize insulin [3], which is the most frequent kind of diabetes, representing 90 to 95 percent of all cases, characterized by insulin resistance and inadequate compensatory insulin production due to pancreatic islet β-cell failure [4,5]. Additionally, the prevalence of type 2 diabetes mellitus (T2DM) is rising, mostly as a result of the sharply rising rates of overweight and obesity [6,7]. Efforts in addressing type 2 diabetes have primarily revolved around enhancing insulin sensitivity, boosting insulin secretion, and impeding or lowering the speed at which glucose is absorbed from the gastrointestinal tract through the means of exercise and dietary approaches aimed at managing obesity [8].

Until now, pharmaceutical treatments for type 2 diabetes consist of biguanides, sulfonylureas, and alpha-glucosidase inhibitors, in addition to semaglutide and tirzepatide, which were recently included in the treatment of type 2 diabetes and obesity. Semaglutide is a GLP-1 analog; tirzepatide is a dual analog of GLP-1 and GIP (glucose-dependent insulinotropic polypeptide) [9].

Nevertheless, these medications possess several limitations in their practical application, as they have been formulated to address symptoms rather than the underlying disease mechanism [10]. As a result, there exists a critical requirement for more effective and secure medications that are reasonably priced, aimed at directly targeting the disease itself, to significantly improve the management of diabetes and its related metabolic complications. Research has shown that protein tyrosine phosphatase 1B (PTP1B) has surfaced as a new and encouraging focal point for therapeutic intervention in the treatment of type 2 diabetes [8,11]. PTP1B protein plays a critical role in the negative regulation of insulin signal transduction pathways. In insulin-sensitive cells, evidence indicates that PTP1B inhibitors increase IR and its substrate phosphorylation, promoting glucose transporters translocation and glucose uptake [12].

While certain PTP1B inhibitors featuring the thiazolidine group have been documented, the majority of these compounds include a significant aromatic component or a charged phosphor tyrosine (pTyr) mimic unit, such as phosphonates, carboxylic acids, and sulfamic acids. These elements have demonstrated limited ability to permeate cell membranes and attain oral bioavailability due to the pronounced negative charge carried by the pTyrmimetics, coupled with their elevated molecular weight. The inherent characteristics of the densely charged active site and the relatively shallow nature of the protein surface surrounding PTP1B pose a significant hurdle for medicinal chemists engaged in the quest for cell-permeable and orally bioavailable PTP1B inhibitors. Consequently, a pressing requirement emerges for the creation of small-molecule PTP1B inhibitors devoid of any charged moieties and with good inhibitory activities that can combat T2DM [1,2,3,4,5,6]. The pharmaceutical industry is currently focused on innovative novel research techniques, such as the ability to anticipate molecules’ activities before their synthesis. Drug design can be sped up by using molecular modeling approaches like QSAR, pharmacophore, Docking, ADMET, and dynamics which are considered to be valuable tools in medicinal chemistry [13,14].

In this study, a set of 27 compounds derived from 5-(substituted benzylidene) Thiazolidine-2,4-dione evaluated as PTP1B inhibitors, was used to describe the 2D-QSAR model developed by the multiple linear regression (MLR) method. Based on a 2D-QSAR model, new compounds were designed and their binding affinity and activity predictions were made with different freely available software/models.

Drug-likeness and ADMET features were used to predict the pharmacokinetic properties of the designed compounds to understand their behavior in the body and predict their overall efficacy and safety profiles. In addition, an analysis of PASS predictions was employed to predict various activities associated with these compounds. In addition, an analysis of PASS predictions was employed to predict various activities associated with these compounds. Furthermore, the stability and reactivity of candidate drugs were also examined using the density functional theory (DFT), and molecular dynamics simulations.

## 2. Results and Discussions

### 2.1. QSAR Results

In this study, the best obtained QSAR model was chosen from among multiple equations using various statistical criteria, such as correlation coefficient R > 0.8 [15], and squared correlation coefficient (R^2^ > 0.6), which is a relative indicator of fit [16]. Standard error of estimate (SEE ˂ 0.3) is an absolute measure of fit quality [17]. Fischer’s value (F), often known as the Fisher ratio represents the ratio of the variance explained by the model to the variance owing to the regression error. The model is statistically significant if the F-test results are high [18,19].

Based on the statistical analyses, the optimized QSAR model for predicting PTP1B inhibition is expressed as follows:

$${pIC}_{50}=-1.169-0.683\times LogP+5.1\times 10^{-3}\times MV+0.157\times MR-0.718\times qN-0.521\times qS+1.038\;qC3\;+2.115\;qC2-1.41\times 10^{-4}\times Tindx-5.446\times 10^{-7}\times Bindx-0.441Clsc$$

where; n = 27; R = 0.942; R^2^ = 0.887; F = 12.584; P = 10; S= 0.223; Q = 4.224; SEE = 0.1

Our optimized QSAR model reveals a correlation between the biological inhibitory activity (pIC_50_) and ten molecular descriptors. The equation indicates that an increase in the values of positive coefficients (MV—Volume, MR—Molecular Refractivity, qC2, and qC3—Mulliken Charges), corresponds to an increase in biological activity, this aligns with the model’s prediction and provides insights into structural features associated with enhanced PTP1B inhibition. Conversely, the negative coefficients for LogP (Coefficient of Partition Octanol/Water), qN, and qS (Mulliken Charges), Tindx (Molecular Topological Index), Bindx (Balaban Index), and Clsc (Cluster Count) suggest that an increase in these parameters leads to a decrease in biological activity, this insight aids in understanding structural features leading to reduced inhibitory effects.

The model demonstrates a strong correlation coefficient (R) of 0.942 and a squared correlation coefficient (R^2^) of 0.887, affirming the robust relationship between descriptors (V, MR, qC2, and qC3) and PTP1B inhibitory activity. The F value for the generated QSAR model significantly exceeds the tabulated F value by a large margin and aligns well to achieve a large regression score. The positive value of the quality factor (Q = 4.224) indicates that this QSAR model has a good predictive potential. The analyses’ accuracy was demonstrated by the low standard error of estimate, which was equal to 0.1.

### 2.2. QSAR Validation

The assessment of a model’s effectiveness relies on a diverse set of statistical analysis criteria, these encompass various criteria to gauge the model’s performance comprehensively, including the coefficient of determination R^2^, and correlation coefficient adjusted coefficient ($R_{adj}^{2}$); these two coefficients enable a comprehensive evaluation of a model’s performance, as well as a combination of other factors such as predicted residual sum (PRESS) factor, sum of the squares of response value (SSY), overall predictive ability ($R_{cv}^{2}$), uncertainty of prediction (S_PRESS_), and predictive square error (PSE). These standards have pivotal roles in measuring the strength and reliability of the model. It leads us to obtain a comprehensive and accurate perspective on the quality and effectiveness of the model [19,20].

The PRESS/SSY ratio can be used to determine estimated confidence intervals for a novel compound’s forecast. This ratio is 0.11, which is less than 0.4, and this confirms that we have a good prediction model [20]. In addition, high values of R^2^_cv_ and R^2^_adj_ (equal to 0.89 and 0.817, respectively) and a low value of SPRESS of 0.09 are the best criteria for qualifying the QSAR model.

The coefficient correlation’s predictive error PE is a measure used to assess the predictive capacity of the resulting model. The generated model met the requirement of R > 6PE, indicating that it has strong predictive power [21] (Table 1).

**Figure 1:**
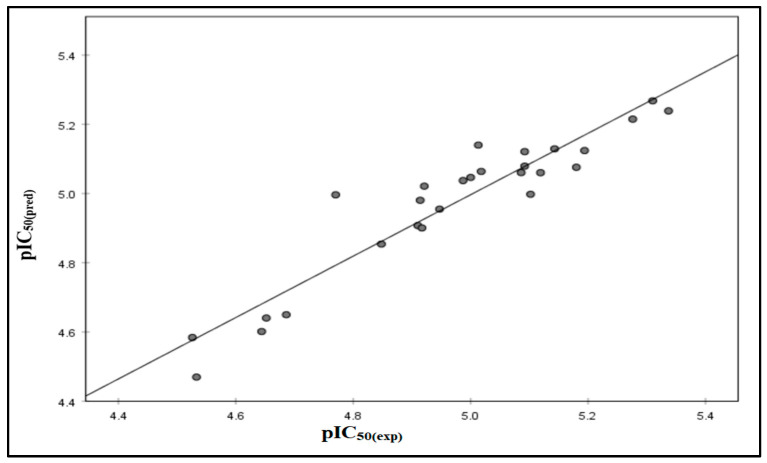
Correlations between the observed activity values and the predicted activity values via the model.

Figure 1 illustrates a linear regression graph comparing expected and experimental biological activity levels. The graphical representation of the model showcases a strong alignment with the empirically observed data, achieving an impressive R^2^ value of 0.848.

**Figure 2:**
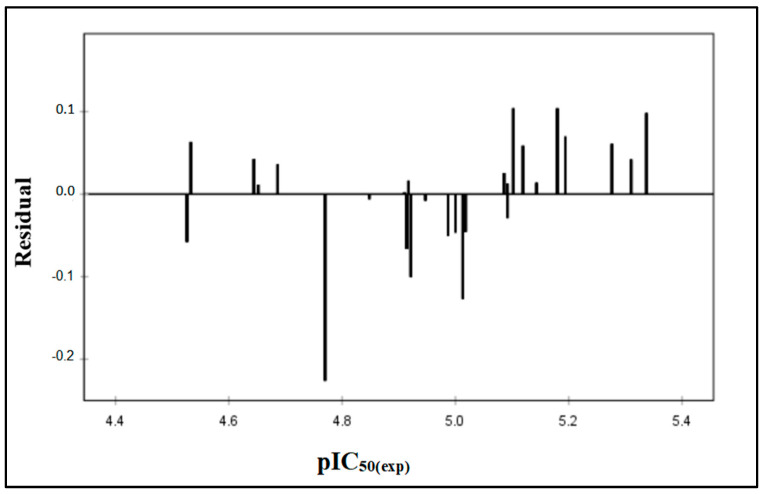
Plots of the residual values against the experimentally observed.

The value of residuals pIC_50_ against the experimental values of the biological activity does not show any systematic error (Figure 2 and Table 2). The distribution of residuals symmetrically around zero implies the absence of systematic error, corroborating the observation made by Jalali-Heravi and Kyani [22]. Thus, the current status of the QSAR investigation indicates the effective applicability of this model in predicting the activity of novel anti-PTP1B compounds within the range of 5-(benzylidene) thiazolidine-2,4-dione derivatives.

The results of the Y-randomization after 50 different randomization trials show the R^2^ and Q^2^ to have low values compared to those of the original model as indicated in Table 3, which shows the robustness of the model. Furthermore, a value of cRp^2^ greater than 0.5 further underscores the high-quality assurance of the model, affirming that its strength is not solely a result of chance.

### 2.3. Applicability Domain

**Figure 3:**
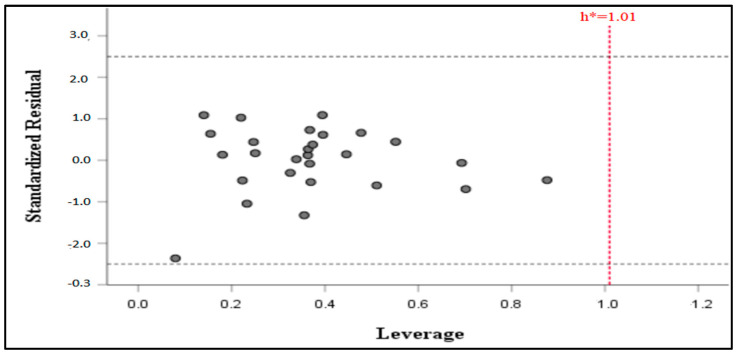
Williams plot illustrating normalized residuals and leverage of the QSAR model.

The purpose of a created QSAR model is for property prediction and will predict effectively only compounds found within the model applicability domain. As shown in Figure 3, all the data sets were found to fall in the domain with no outliers. The threshold (h*) of the model was calculated to be h* = 1.01, and no compound was found beyond the threshold, which points to the reliability of the MLR-QSAR model’s predictions.

### 2.4. Design of New Compounds

The objective of this work was to design ligands that have improved activity and binding affinity, based on the structural properties and properties of ligand **7e(ref)**, which was used as a template (the most active molecule in the database), as shown in Table 10.

We noticed that the QSAR model showed a reversal relationship between pIC_50_ and LogP, i.e., decrease in hydrophobicity is associated with an increased biological activity. In light of this, the value of the LogP of all designed ligands is less than the LogP of ligand **7e(ref)** (5.247).

On the other hand, there is a proportional effect between molar refractivity and volume on biological activity.

On this basis, we proposed and designed seven derivatives by modifying the chemical structure of molecule **7e(ref)**. This involved reducing the hydrocarbon groups and incorporating polar substituents (–OH, –OCH_3_, etc.) in the position R_1_ and large polar substituents group (Pyrrolidinyl, piperidinyl, hydroxypropyl, etc.) in the position R_2_, to increase the hydrophilicity and volume of the compounds.

**Figure 4:**
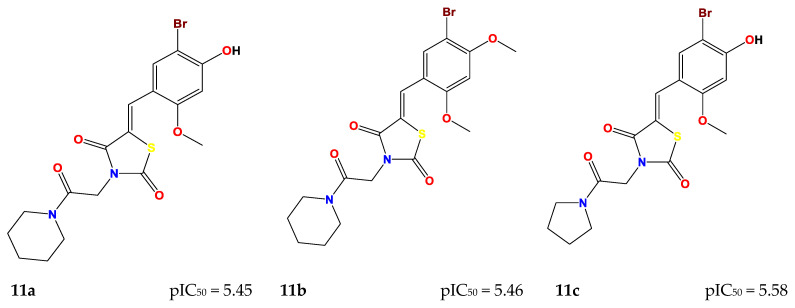

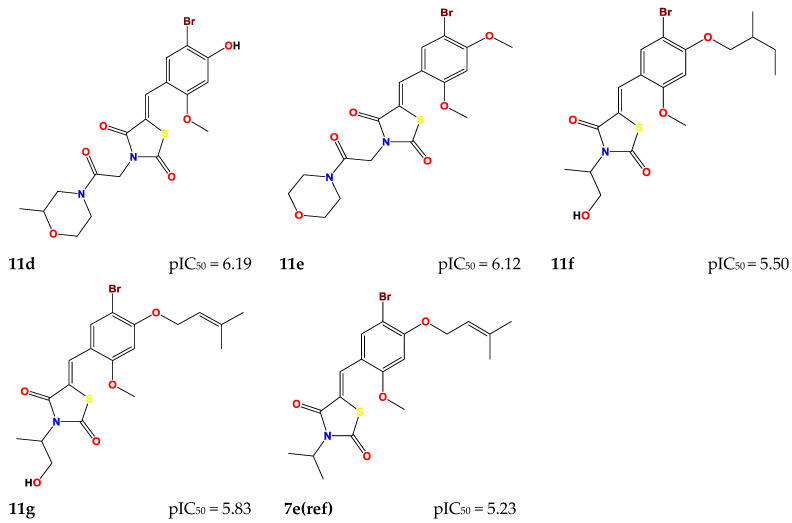
Newly designed compounds and their predicted pIC_50_.

These seven compounds (**11a**–**g**) showed a higher pIC_50_ compared to the molecules of the series, especially compound **7e(ref)**, which was the most active compound (pIC_50_ = 5.337). The chemical structures of designed compounds and their predicted pIC_50_ values are shown in Figure 4.

### 2.5. Molecular Docking

#### 2.5.1. Docking Validation Protocol

**Figure 5:**
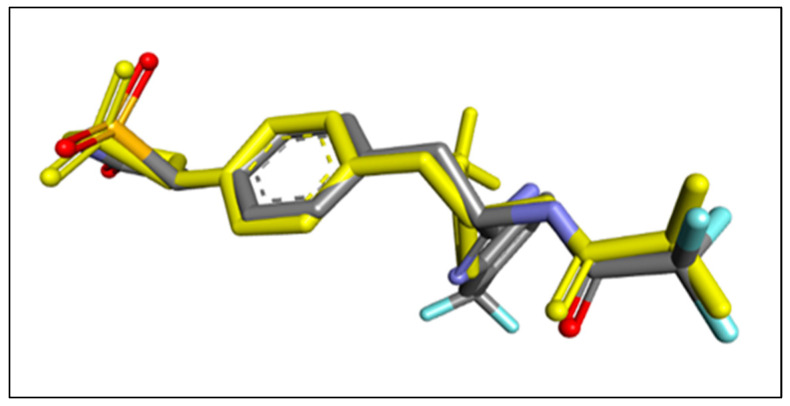
3D visualizations of the re-docking pose along with RMSD value of 1.78 Å. The re-docked pose is represented in yellow, while the original pose is indicated in blue.

The docking approach was confirmed by re-docking the co-crystalized ligand IZE at the protein (PDB: 2cng). This verification process involves aligning the docked ligand with the ligand bound in the protein, as illustrated in Figure 5. To quantify the alignment, the RMSD was computed via the online tool DockRMSD (https://zhanggroup.org/DockRMSD/) (accessed 15 November 2023). [23,24], resulting in a value of 1.78 Å. This value underscores the remarkable consistency between the docked ligand and the co-crystalized ligand orientations. Significantly, the RMSD value falls within the dependable threshold of less than 2 Å [25], providing strong affirmation of the accuracy of the docking protocol.

#### 2.5.2. Analysis of Interactions between Newly Designed Ligands and Protein Active Site

Before beginning the molecular docking procedure, the structures of the designed compounds were optimized using Gaussian 09W software utilizing the DFT/B3LYP technique with the base 6-311G+ (d, p). We docked the reference ligand to the protein receptor to validate the binding energy of ligand−protein interactions. The results show that the binding affinity values of the seven novel suggested inhibitors range between −7.30 and −8.18 kcal/mol, while the binding affinity value of the reference ligand is −7.19 kcal/mol. In addition, these findings show that the proposed inhibitors may be for stable complexes with the protein and that the created 2D-QSAR model has a high predictive potential. Table 4 displays the docking modeling findings for each designed inhibitor and the reference ligand.

Molecular docking revealed that the reference compound **7e(ref)** formed a hydrogen bond TYR46 with the bromine attached to the benzene ring at a distance of 3.16 Å, Additionally, it formed a carbon hydrogen and electrostatic bond with the thiazolidine ring and ASP48, with distances of 3.17 Å and 3.03 Å, respectively, and alkyl and π-alkyl bondsALA217, CYS215, VAL49, and ARG221.

Compound **11c** attained the highest binding energy by participating in various interactions with the protein. Specifically, it formed a hydrogen bond with ASP181, involving the hydrogen of the alcohol attached to the benzene ring at a distance of 1.81 Å. It also established two carbon-hydrogen bonds with ASP48 and SER216, at distances of 3.59 Å and 3.40 Å, respectively. Additionally, electrostatic interactions took place with ASP48, and pi-sigma interactions occurred with TYR46 and ALA217. Furthermore, there were alkyl-type hydrophobic interactions involving ALA217, CYS215, VAL49, and ARG24, in addition to a pi–pi stacking bond with TYR46. The second-best recorded compound was 11a, where a noticeable similarity was observed between it and compound **11c** in all interactions, except for the absence of the alkyl bond with ARG24 residues, and differences in the lengths of formed bonds, ranking compound **11b** as the third-best interacting compound with a protein. Notably, it formed two carbon–hydrogen bonds with TYR46 and ASP48, at distances of 2.88 Å and 2.98 Å, respectively. Furthermore, a pi-sigma interaction with ALA217 was observed, alongside an electrostatic interaction with ASP48. This was accompanied by hydrophobic interactions of the alkyl type and Pi–alkyl interactions with residues ALA217, CYS215, and LYS120, in addition to a Pi–sulfur bond with PHE182. Next is compound **11d**. This compound distinguished itself by forming two hydrogen bonds, one with ASP181 and another with TYR20 at distances of 1.81 Å and 3.14 Å, respectively. Additionally, it established a carbon–hydrogen bond with the amino acid SER216 at a distance of 3.70 Å. An electrostatic interaction with ASP48 was also observed, along with a pi–sigma interaction with ALA217. It also engaged in alkyl-type and Pi–alkyl interactions with amino acids ALA217, ILE219, and TYR46, as well as a pi–pi stacking interaction with PHE182.

Ranked fifth, compound **11F** forms crucial interactions with the protein, creating a hydrogen bond with ASP48 at a distance of 1.93 Å and a hydrogen–carbon bond with SER216 at a distance of 3.69 Å. Additionally, it engages in electrostatic interaction with ASP48. Furthermore, it participates in alkyl and Pi-alkyl type interactions with ALA217, CYS215, VAL49, and ARG221, and establishes pi–pi stacked and pi–pi shaped interactions with Tyr46 and Phe182, respectively. Next is compound **11e**, which has formed a hydrogen bond with TYR20 at a distance of 3.27 Å, as well as two hydrogen–carbon bonds with TYR46 and ASP181 at distances of 3.29 Å and 2.78 Å, respectively. Additionally, it exhibits an electrostatic interaction with ASP48, along with alkyl and Pi-alkyl type interactions involving residues ALA217, TYR46, LYS120, ILE219, and CYS215. Furthermore, it engages in a pi–pi stacked interaction with Phe182.

Among the designed compounds, the one with the lowest binding energy is observed to be compound **11g**. In this case, it forms a hydrogen bond and a hydrogen-carbon bond with the amino acid ASP48 at distances of 2.20 Å and 3.46 Å, respectively. Additionally, there is an electrostatic interaction with ASP48. It also interacts through pi-sigma interactions with ALA217 and Phe182, in addition to engaging in alkyl and Pi-alkyl type interactions with the amino acids ALA217, CYS215, ILE219, ARG221, and VAL49. Furthermore, it forms pi–pi stacked and pi–pi shaped interactions with Tyr46 and Phe182, respectively.

**Figure 6:**
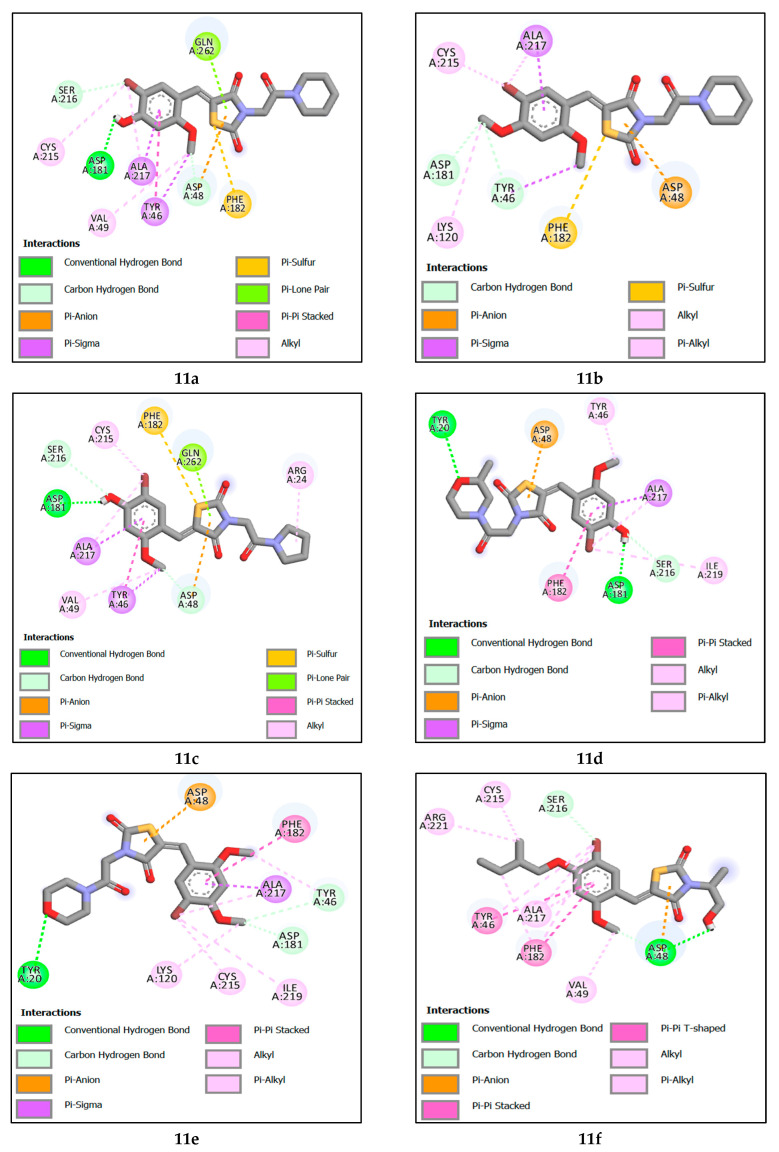

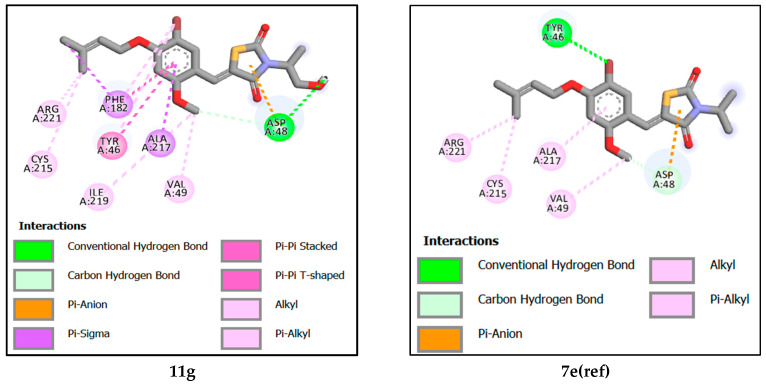
The 2D molecular interactions of designed compounds **11a**, **11b**, **11c**, **11d**, **11e**, **11f**, **11g** and reference ligand **7e(ref)** towards the 2CNG protein.

This study found that hydrogen bond interactions are responsible for inhibiting the biological function of tyrosine phosphatase 1B. The amino acids in the receptor of tyrosine phosphatase 1B which interacted with all designed inhibitors by hydrogen bonds, were ASP48, ASP181, SER216, TYR46, and TYR20 at a distance between 1.80 and 3.96 Å. In addition, the reference molecules interact with the amino acids ASP48 and TYR46 with a distance between 3.16 and 3.17 Å. For hydrophobic interaction, the proposed tyrosine phosphatase 1B interacts with the amino acids ALA217, TYR46, CYS215, VAL49, LYS120, ARG24, PHE182, ILE219 and in distances between 3.12 and 4.88 Å. While the reference compound interacts with the amino acids at a further distance. Finally, all proposed molecules including the reference ligand interact with the ASP48 amino acid in the same way by electrostatic interaction and at the same distance. Figure 6 shows the binding interactions of the seven designed molecules and the reference ligand with the tyrosine phosphatase 1B receptor. Furthermore, this similarity shows the inhibitory power of the proposed molecules for the biological activity of tyrosine phosphatase 1B receptor.

Molecular docking analysis reveals that the studied ligands were surrounded by ASP48, ASP181, SER216, ALA217, TYR46, CYS215, VAL49, ARG24, TYR20, ASP48, PHE182, GLN262, LYS120, ARG221, ILE219 and ARG47 within the binding site of PTP1B, and therefore covering a larger area of interactions. Mainly, the active-site Cys215 is known for nucleophilic behavior due to the thiol group (-SH) in its side chain [8]. Our findings on the interactions between the seven designed molecules and the active site of PTP1B are consistent with those of Sónia Rocha et al. [4].

### 2.6. ADME-Tox Prediction and Bioavailability

Pharmacokinetics knowledge is critical in the drug design process. In addition, solubility and permeability to biological membranes are two of the most critical parameters determining medication effectiveness. LogP was calculated for all the designed compounds, and all values were within the permitted range from 1.97 to 3.63. These findings suggest that all of the proposed molecules have excellent permeability into biological membranes. In terms of solubility in the aqueous media, all of the proposed compounds revealed LogS values between −5.12 and −3.89, ensuring their solubility in the aqueous medium is completely absorbed, in contrast to the reference molecule, whose aqueous solubility is minimal. The bioavailability score of all proposed molecules was 0.55, indicating that they had high gastrointestinal absorption. On the other hand, all designed molecules are simple to synthesize in a chemical laboratory, which simplifies their evaluation in vitro and in vivo (with synthetic accessibility less than 4.61). Finally, all of these compounds agree with Lipinski’s criteria and none of them contain any structural fragments recognized as PAINS. Table 5 reflects all of the information collected via SwissADME database.

**Figure 7:**
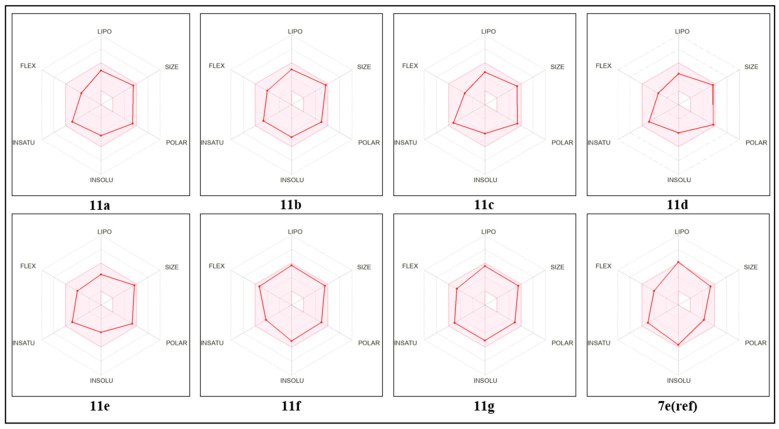
Bioavailability radar for proposed molecules and a reference compound, depicting the FLEX (rotatable bond flexibility), LIPO (lipophilicity), SIZE (molecular weight), POLAR (polarity), INSOLU (insolubility), and INSATU (insaturation) parameters.

Bioavailability radar is available for rapid drug-likeness testing. Six physical and chemical features are taken into account by the bioavailability radar. Size, lipophilicity, polarity, solubility, saturation, and flexibility represent these characteristics. If the graph is in this pink location, it is a drug-like compound for the compound. Figure 7 describes the compound’s bioavailability radar plots. We can confirm that all of these compounds have high bioavailability and an acceptable pharmacokinetic profile based on these radars.

To lower the cost and resources spent on the drug’s preclinical and clinical studies, toxicity is crucial. It indicates the extent to which a chemical may affect an organism or a substructure of an organism [26]. The hepatotoxicity, carcinogenicity, immunotoxicity, mutagenicity, and cytotoxicity predictions for the novel proposed (Table 6) were inactive, suggesting that these compounds may be relatively less toxic. Where all the proposed compounds had an LD_50_ value between 1000 and 1400 mg/kg, and a toxicity class equal to four.

Employing VEGA QSAR, we evaluated mutagenicity (Ames test), skin irritation, plasma protein binding, P-Glycoprotein activity, and total body elimination half-life. Table 7 represents all results obtained via VEGA QASR. The acquired results suggest none of the proposed compounds are a danger to biological organs, indicating their effectiveness and safety.

### 2.7. Biological Activities Using PASS

To analyze the results, the scoring system used was as follows: activity with higher probability of occurrence (Pa > 0.7), probable probability of occurrence (0.5 < Pa < 0.7) and unlikely probability of occurrence (Pa < 0.5). Thus, it predicted which pharmacological activities are very likely, probable or unlikely for molecules previously tested in in vivo experiments.

The designed compounds **11a**–**11g** show a large spectrum of pharmacological activity principally, anaphylatoxin receptor antagonist (Pa > 0.7), thiol protease inhibitor (0.7 > Pa > 0.5) and other activities less than 0.5 such as antibacterial, calcium channel N-type blocker, amyloid beta precursor protein antagonist, HCV NS3-helicase inhibitor, antidiabetic, etc.

Table 8 reveals important anticipations of the pharmacological potential directly associated with diabetes mellitus of newly designed ligands. Remarkably, the observed effects include the inhibition of protein-tyrosine phosphatase 1B, protein-tyrosine phosphatase 1B, and general protein-tyrosine phosphatase activity. Moreover, antidiabetic, antidiabetic symptomatic, and antidiabetic (type 2) activities.

As shown in Table 8, the inhibition of protein-tyrosine phosphatase ranges between 0.344–0.420, indicating high Pa values compared to other activities. Additionally, protein-tyrosine phosphatase type beta inhibitors exhibit the lowest probability of inactive for all compounds. Furthermore, all compounds show a very weak probability Pa for antidiabetic (type 2) activity except for two compounds **11e** and **11d**.

### 2.8. Frontier Orbital Energies and Global Reactivity Parameters Using DFT

Density functional theory (DFT) was utilized to explore the stability and chemical reactivity of the designed compounds. Table 9 presents the results, the values of the energetic gap (Eg) ordered the studied compounds from the least reactive to the most, as follows: **11b** < **11g** < **11e** < **11a** < **11d** < **11c** < **11f**. Therefore, the hardness values show the same tendency of Eg, in contrast, the softness values have the opposite order according to the following: **11f** > **11c** > **11d** > **11a** > **11e** > **11g**. Thus, compound **11f** has the highest reactivity while compound 11b has the lowest reactivity and is more stable than the others.

**Figure 8:**
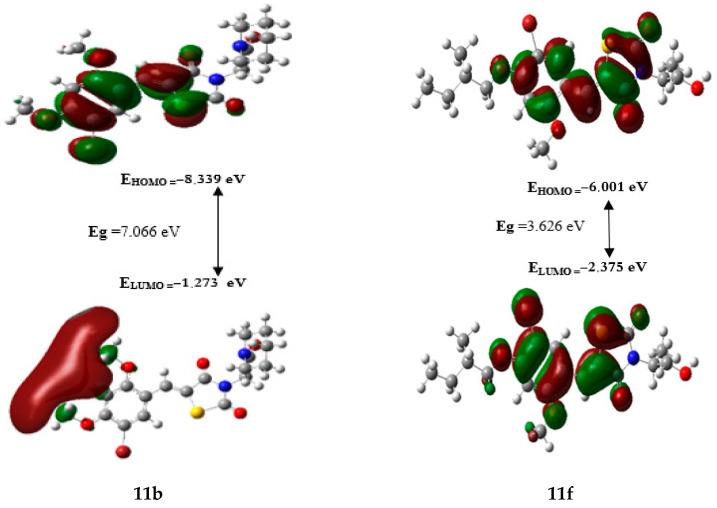
HOMO and LUMO molecular orbitals of compounds **11b** and **11f**.

A general relationship exists between hardness, softness and Eg, which is characterized by a low kinetic stability (low Eg) and a high chemical reactivity (low η and high δ). The HOMO-LUMO gaps of compounds 11fis represented in Figure 8. Where, the red areas represent the attractive potential and the green areas represent the repulsive potential, both of which play a crucial role in chemical reactivity.

Additionally, Table 9 shows that every compound has a negative chemical potential value, indicating that the compound is stable.

The electrophilicity index (**ω**) explains the ability to accept electrons from the external environment, particularly when expressed positively [27,28,29]. Based on that, compound **11e** is regarded as an excellent electrophile due to its low value of E_LUMO_ (−2.627 eV) and high value of ω (5.412 eV).

### 2.9. Molecular Dynamics Results

#### 2.9.1. Root Mean Square Deviation Analysis (RMSD)

**Figure 9:**
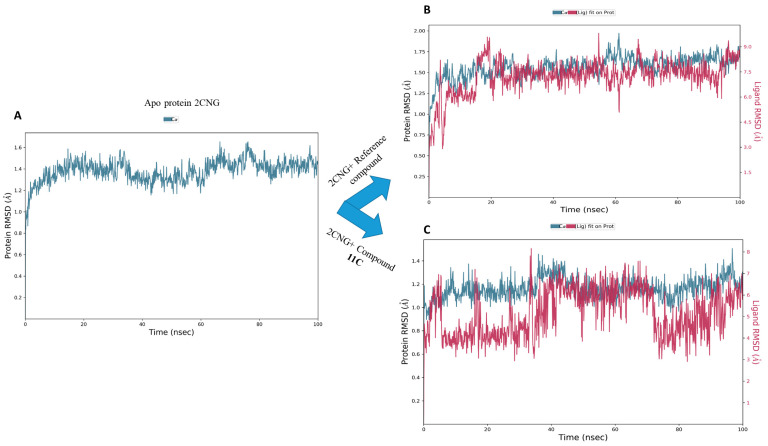
The root mean square deviation plots of the Apo protein (**A**), 2CNG, and the complexes **11c**-2CNG (**B**), co-crystalized ligand-2CNG (**C**).

The analysis of the root mean square deviation (RMSD) serves as a pivotal indicator of the system’s stability during molecular dynamics simulation [30]. In the investigation of the backbone atoms of the protein, both in its unbound state and in the presence of compound **11c** and the co-crystallized ligand, the RMSD plots were examined over the simulation duration. As depicted in Figure 9, equilibrium was achieved at approximately 20 ns for both the free system and the bonded system, revealing an average protein RMSD of 1.5 Ǻ. This observation underscores the inherent stability of the protein, even in the dynamic context of interactions with the studied compounds throughout the simulation period. Notably, for compound **11c**, the ligand RMSD exhibited an average value of 3.5 Ǻ, with minimal fluctuation in alignment with the protein’s RMSD. Similarly, in the case of the co-crystallized ligand, both the protein and ligand RMSD trajectories demonstrated alignment, with a slightly higher average RMSD of 5.5 Ǻ. These results collectively suggest that the introduction of compound **11c** and the co-crystallized ligand does not significantly compromise the overall stability of the protein, as evidenced by the relatively consistent RMSD values and aligned trajectories of the protein–ligand throughout the simulation period.

#### 2.9.2. Root Mean Square Fluctuation (RMSF) Analysis

**Figure 10:**
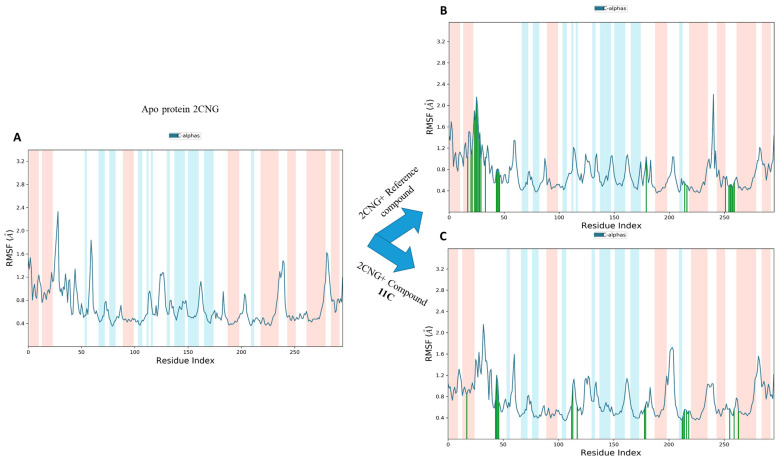
The RMSF plots of the Apo protein (**A**), 2CNG-Co-crystallized compound (**B**), and 2CNG-**11c** (**C**).

The analysis of root mean square fluctuation (RMSF) provides valuable insights into the dynamic behavior of amino acid residues within drug–target complexes [31]. High RMSF values typically indicate increased flexibility in amino acids, while low values suggest stability in specific receptor regions. In our study, as depicted in Figure 10, RMSF curves revealed that the majority of amino acid residues in the drug–target complexes exhibited slight fluctuations, generally remaining below 2.4 Ǻ. This finding suggests noteworthy stability in these residues, emphasizing the overall structural integrity of the protein within the complex. When compared to the RMSF of the apo-protein, this observation gains significance. The relatively low RMSF values indicate that the introduction of these active components (compound **11c** and the co-crystallized compound) does not induce disruptive fluctuations in amino acid residues.

#### 2.9.3. Protein–Ligand Interaction Analysis

**Figure 11:**
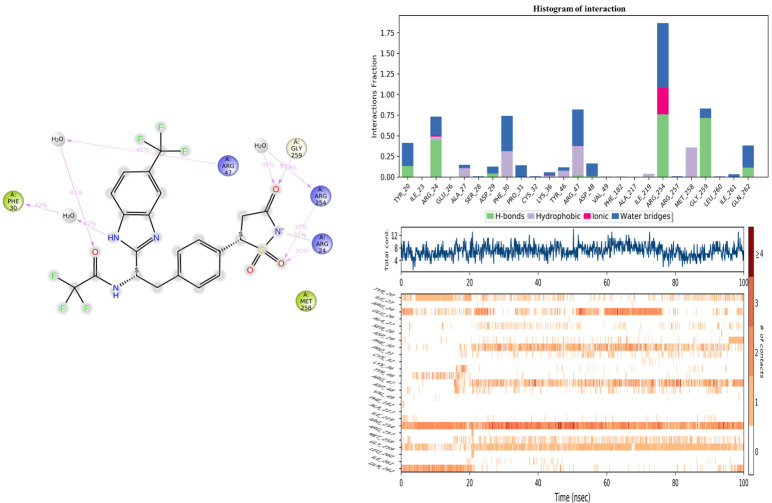
Histogram of interaction, and the number of contacts between 2CNG and the co-crystallized ligand.

**Figure 12:**
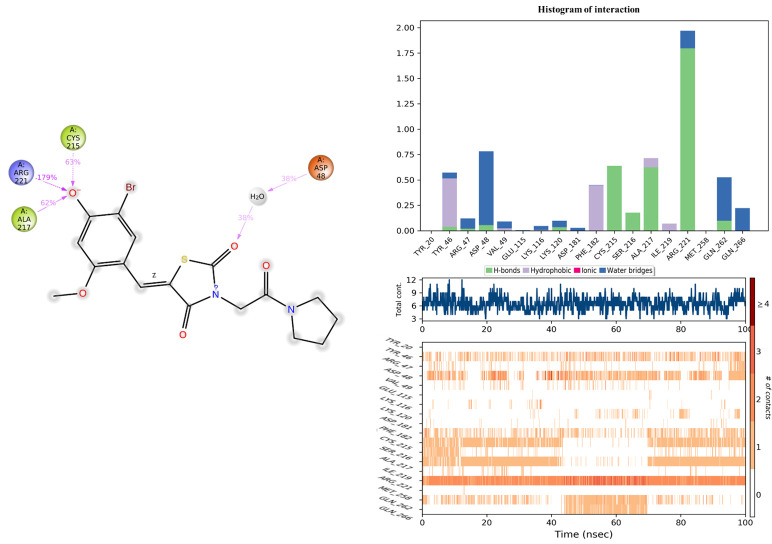
Histogram of interaction, and the number of contacts between 2CNG and the **11c** compound.

The assessment of the protein–ligand interaction fraction provides crucial insights into the binding characteristics between the targeted proteins and the considered candidates. Notably, compound **11c** demonstrated interactions with 18 residues, while the co-crystallized compound engaged with 25 residues. These interactions encompassed a diverse array of bonding types, including hydrogen bonds, hydrophobic interactions, ionic interactions, and water bridges. The identification of key residues with substantial fractions and a high number of contacts further illuminates the specificity and significance of these interactions. For compound **11c**, pivotal residues such as ARG221, PHE182, CYS215, ALA217, TYR46, and ASP48 were highlighted, indicating the critical role of these amino acids in mediating the compound’s binding to the target protein. Similarly, the co-crystallized compound exhibited significant interactions with key residues, notably ARG254, ARG47, and GLY259. These findings underscore the nuanced and specific nature of the interactions, with each compound establishing connections with distinct amino acid residues. The diversity of interaction types and the identification of key residues contribute to a comprehensive understanding of the binding mechanisms, offering valuable insight into the drug–target interactions for the pursuit of therapeutic efficacy (Figure 11 and Figure 12).

## 3. Materials

### 3.1. QSAR Analysis

The experimental study provided us with information on 27 derivatives of 5-(substituted benzylidene) thiazolidine-2,4-dione, including the values of its inhibited activity (IC_50_) against the protein tyrosine phosphatase 1B (PTP1B) [6]. The values have been converted to a logarithmic decimal scale (pIC_50_ = −log10 IC_50_) [32], as shown in Table 10.

### 3.2. Computation of Molecular Descriptors

Quantitative structure–activity relationships (QSAR) modeling serves as a potent avenue for investigating and capitalizing on the interplay between chemical structure and its biological effects, contributing to the advancement of novel drug candidates. The QSAR methodology can be broadly defined as the utilization of data analysis techniques and statistical principles to construct models that can precisely anticipate the biological activities or properties of compounds, based on their structures [33,34]. In our work, we tried to construct a QSAR model of a series of 5-(benzylidene) thiazolidine-2,4-dione derivatives having inhibitory biological activity for PTP1B. We calculated 19 distinct molecular descriptors of the examined series that correspond to different classes (1D, 2D, 3D) to create a linear mathematical model.

Firstly, the examined 27 compounds geometries were optimized using the DFT/B3LYP approach with the 6-311G+ (d, p) basis by the Gaussian 09 program [35,36]. It was confirmed to be a global minimum for each compound by not having any imaginary frequency. From Gaussian calculation, we can obtain reasonably accurate values of electronic and quantum descriptors (E_HOMO_, E_LUMO_, ET, and charges of atoms). Then, the topological, physicochemical descriptors were calculated after optimization using the Chem3D V15.1 [37], HyperChem (8.0.8) [38] SwissADME [39], and pkCSM [40] (Table 11 and Table 12).

Finally, we used the multiple linear regression (MLR) method to predict the biological activity of the data set as a linear function of the structural properties, which correlates the ordinate y (pIC_50_) as a dependent variable to the molecular descriptors xi [17,18,19,20,21,22,23,24,25,26,27,28,29,30,31,32,33,34,35,36,37,38,39,40,41]. The analysis was conducted using SPSS version 19 for Windows [42]. All the calculations were performed using a 64-bit Operating System on Intel^®^ Dual Core Processor AMD Ryzen 9 5900HS with Radeon Graphics 3.30 GHz of memory and 250 GB scratch disk space.

### 3.3. Y-Randomization

Y-randomization serves as a crucial technique to confirm the robustness and reliability of a developed QSAR model, ensuring it is not merely a result of chance [43]. This test is instrumental in preventing the occurrence of random correlations between molecular descriptors (X values) and their corresponding biological activities (Y values) in the obtained model. The Y-randomization test involves the random distribution of experimental properties/activity values onto the descriptors of the original model, generating new models as a result [44]. To deem the QSAR model acceptable and to ascertain that it was not fortuitously obtained, the average random correlation coefficient (*R_r_*^2^) of these randomly constructed models should be lower than the correlation coefficient (*R*^2^) of the original non-random model [45]. Additionally, the model must pass the Y-randomization test by having a cRp^2^ value greater than 0.5, which is calculated using the mathematical formula as follows:

$${cRp}^{2}=R\times {((R}^{2}{)-(Average\;R_{r})}^{2}{)}^{1/2}$$

### 3.4. Applicability Domain

QSAR models have been established using a limited set of compounds that does not encompass the entirety of chemical space. The domain of applicability (DA) delineates the specific region within the chemical space where the QSAR model can accurately predict new compounds. Therefore, assessing the DA is crucial to ensure the dependable utilization of QSAR models [1]. Building the model applicability domain involves plotting the leverages of each compound against their respective standardized residuals. The diagonals of the hat matrix, $H=X{\left(X^{T}X\right)}^{-1}X^{T}$ produce the leverages for each of the compounds [46], where *X* is the descriptor matrix and *X^T^* is the transpose of *X*. The domain has warning leverage, h^∗^ = 2.5(K + 1)/n, where k and n represent the numbers of the training set and model descriptors, respectively. Compounds beyond the warning leverage $\left(h>h^{*}\right)$ are chemically different from the training set compounds and are considered outliers and not reliably predicted by the model [47,48].

### 3.5. Molecular Docking

Molecular docking has emerged as a crucial approach in the field of drug discovery and development [49,50] and it is considered as a prominent method for explaining the receptor–ligand interactions valid in drug discovery. This method was used to predict the placement, binding affinity, and inhibitor activity of co-crystallized ligands in the binding pocket of PTP1B [51]. The 3D crystal structure of PTP1B was downloaded from the Protein Data Bank (PDB ID: 2cng). The process of molecular docking simulation was conducted using Autodock 1.5.7. Prior to docking, the protein structures were cleaned by removing water molecules, ligands, and cofactors. The Gasteiger charges were then computed, and polar hydrogens were inserted, while non-polar hydrogens were combined using Autodock [52,53]. The Auto Grid technique was used to construct a 3D grid that would analyze the energy of complex ligand–protein interactions. The grid maps had a default grid space size of 0.375 and were set to 40 in all directions (X, Y, and Z axes). The central grid box in the protein was found at (2.124, 9.760, and 46.315) by the ligand location in the protein. The molecular docking studies were performed using the Lamarckian Genetic Algorithm (LGA), which is known for being one of the best docking methods available in Autodock [52,53,54]. To visualize binding modes, the type of interactions, and exhibit the surfaces of cavities, Biovia Discovery Studio software 2021 was used [55].

### 3.6. Molecular Dynamics Simulation

We employed molecular dynamics (MD) simulations to investigate the dynamics behavior of compound 11c and the co-crystallized ligand in complex with tyrosine-protein phosphatase non-receptor Type 1 (PDB ID: 2cng) for 100 nanoseconds [56]. Additionally, simulations were conducted for the apo-protein. The Maestro-Desmond software (Version 12.5.139) program, employing the OPLS3e force field, was utilized for the simulations [57,58]. The system was modeled in a periodic cubic box with a TIP3P water solvation system at 300 K [59]. To ensure system neutrality, salt ions (Na^+^ and Cl^−^) were added at precise concentrations. Temperature and pressure equilibration were achieved using a Nose–Hoover chain thermostat, maintaining a pressure of 1.01 bar [60]. MD simulations were initially performed for 1 ns under the NVT ensemble at 300 K, followed by 100 nanoseconds under the NPT ensemble. Subsequently, the MD trajectory results were analyzed to determine the root mean square deviation (RMSD), root mean square fluctuation (RMSF), and protein–ligand interactions.

### 3.7. In Silico and ADME and Drug-Likeness Prediction

A drug’s success can be gauged, not simply through its effectiveness but also by a satisfactory ADMET identity. While there are several high-throughput in vitro ADMET screens accessible, having the ability to predict a number of these features occurs in silico [61,62]. The drug-likeness prediction and pharmacokinetics properties of the newly designed compounds were calculated using free web tools including the SwissADME web server [63] and the ProTox-II platform, which was implemented to evaluate the toxicity of the selected compounds [64]. Other essential properties, such as developmental toxicity, skin irritation, plasma protein binding, P-Glycoprotein activity, and whole-body elimination half-life, were evaluated using VEGA QSAR [65].

### 3.8. PASS Prediction

PASS (Prediction of Activity Spectra for Substances) is a software product designed as a tool for evaluating the general biological potential of an organic drug-like molecule. PASS provides simultaneous predictions of many types of biological activity based on the structure of organic compounds. Thus, PASS can be used to estimate the biological activity profiles for virtual molecules, before their chemical synthesis and biological testing [66,67].

The structures of our designed compounds were drawn with ChemDraw 15.1, and then converted into their SMILE format and used to calculate or predict biological spectrum using the PASS online version (http://www.way2drug.com/passonline) (accessed 29 November 2023). The calculated results are presented as Pa (probability for active compound) and Pi (probability for inactive compound). Here, Pa > Pi is considered on a scale of 0.000 to 1.000 and in general, Pa + Pi ≠ 1 [68]. 

### 3.9. Assessing Chemical Reactivity through DFT Calculations

In order to assess the stability and chemical reactivity of the designed compounds, we used Gaussian 09 to perform calculations using the DFT/B3LYP/6–311++G(d)(p) basis set. These calculations involved the determination of the highest occupied molecular orbital (HOMO) and lowest unoccupied molecular orbital (LUMO) energies.

The HOMO and LUMO energies are used for the determination of global reactivity descriptors such as chemical potential (μ), hardness (η), softness (δ), and electrophilicity (ω). These parameters are calculated by the following equations [27,28,29,30,31,32,33,34,35,36,37,38,39,40,41,42,43,44,45,46,47,48,49,50,51,52,53,54,55,56,57,58,59,60,61,62,63,64,65,66,67,68,69]:

$$E_{g}=E_{LUMO}-E_{HOMO}$$

$$\mu =\frac{E_{LUMO}+E_{HOMO}}{2}$$

$$ɳ=\frac{E_{LUMO}-E_{HOMO}}{2}$$

$$\omega =\frac{\mu^{2}}{2ɳ}$$

$$\delta =\frac{1}{2ɳ}$$

## 4. Conclusions

In conclusion, our study employed a comprehensive quantitative analysis through the application of the (QSAR) on a dataset comprising 27 compounds of 5-(substituted benzylidene) thiazolidine-2.4-dione derivatives, known for their inhibitory activity against PTP1B. Utilizing the MLR method, we successfully developed a predictive 2D-QSAR model, incorporating 10 structural descriptors, to elucidate the biological activity (pIC_50_) of the investigated compounds. The established QSAR model demonstrated a robust predictive capacity, as evidenced by the substantial agreement between the experimentally determined and predicted values of pIC_50_. This suggests the model’s efficacy in forecasting the inhibitory activity of novel compounds within this specific category of derivatives targeting PTP1B. We designed seven new 5-(substituted benzylidene) thiazolidine-2.4-dione derivatives as PTP1B inhibitors. Molecular docking studies affirmed the heightened stability of these newly conceived compounds within the PTP1B receptor, surpassing the reference compound **7e(ref)** in activity within the dataset. Furthermore, our designed compounds adhere to drug-likeness rules, positioning them as potential candidates for oral drug development. ADMET evaluation underscored their pharmacological activity, with all compounds exhibiting significant implications for potential therapeutic interventions associated with diabetes mellitus. Notably, the molecular dynamic simulation conducted on the highest-ranked compound, **11c**, validated its stability, further supporting its potential as a promising candidate for PTP1B inhibitions. This collective evidence reinforces the credibility and applicability of our findings, paving the way for the development of novel therapeutics in the realm of diabetes mellitus.

## Figures and Tables

**Table 1:** Cross-validation parameters.

R^2^_adj_	R^2cv^	S_PRESS_	SSY	PRESS	PRESS/SSY	PE	6PE
0.817	0.89	0.096	1.30	0.147	0.11	0.015	0.09

**Table 2:** Experimental and predicted pIC_50_ values of the 27 5-(substituted benzylidene) thiazolidine-2,4-dione derivatives using the QSAR model.

Comp	pIC_50(Exp)_	pIC_50(Pred)_	Residual
**6a**	4.526	4.584	−0.058
**6b**	4.947	4.955	−0.008
**6c**	4.848	4.854	−0.006
**6d**	5.092	5.079	0.013
**6e**	5.276	5.215	0.061
**6f**	5.310	5.268	0.042
**6g**	5.119	5.06	0.059
**7a**	4.652	4.64	0.012
**7b**	5.092	5.121	−0.029
**7c**	5.086	5.06	0.026
**7d**	4.770	4.996	−0.226
**7e(ref)**	5.337	5.239	0.098
**7f**	4.910	4.908	0.002
**7g**	5.000	5.047	−0.047
**7h**	4.987	5.037	−0.05
**7i**	4.917	4.901	0.016
**7j**	5.102	4.998	0.104
**7k**	5.013	5.14	−0.127
**7m**	5.180	5.076	0.104
**12**	4.914	4.98	−0.066
**13d**	4.644	4.601	0.043
**13e**	4.686	4.65	0.036
**14b**	5.194	5.124	0.07
**14d**	4.921	5.021	−0.1
**14e**	5.018	5.064	−0.046
**14f**	5.143	5.129	0.014
**14g**	4.533	4.47	0.063

**Table 3:** Results of the Y-Randomization test.

Model	R	R^2^	Q^2^	Model	R	R^2^	Q^2^
Original	0.942	0.887	0.631	Original	0.942	0.887	0.631
Random 1	0.480	0.230	−1.662	Random 26	0.614	0.376	−2.392
Random 2	0.425	0.181	−3.135	Random 27	0.654	0.428	−0.496
Random 3	0.671	0.451	−0.396	Random 28	0.602	0.362	−0.660
Random 4	0.635	0.403	−0.183	Random 29	0.687	0.472	−0.434
Random 5	0.595	0.354	−1.660	Random 30	0.546	0.299	−1.066
Random 6	0.771	0.594	−0.557	Random 31	0.705	0.497	−0.220
Random 7	0.629	0.396	−0.771	Random 32	0.453	0.205	−1.060
Random 8	0.565	0.320	−0.734	Random 33	0.598	0.358	−1.243
Random 9	0.431	0.186	−0.583	Random 34	0.565	0.320	−1.367
Random 10	0.651	0.424	−0.874	Random 35	0.657	0.431	−0.602
Random 11	0.719	0.517	−0.429	Random 36	0.563	0.317	−1.031
Random 12	0.534	0.285	−1.298	Random 37	0.703	0.495	−0.175
Random 13	0.554	0.307	−0.693	Random 38	0.555	0.308	−0.931
Random 14	0.764	0.584	−0.294	Random 39	0.604	0.365	−1.955
Random 15	0.545	0.297	−1.552	Random 40	0.453	0.205	−2.432
Random 16	0.761	0.579	−0.193	Random 41	0.574	0.329	−1.018
Random 17	0.454	0.206	−1.148	Random 42	0.497	0.247	−1.088
Random 18	0.597	0.357	−0.916	Random 43	0.642	0.413	−0.917
Random 19	0.682	0.465	−0.683	Random 44	0.692	0.479	−0.399
Random 20	0.676	0.457	−0.448	Random 45	0.405	0.164	−1.100
Random 21	0.450	0.203	−1.006	Random 46	0.398	0.158	−1.967
Random 22	0.558	0.311	−0.927	Random 47	0.627	0.394	−0.661
Random 23	0.678	0.460	−1.264	Random 48	0.642	0.412	−0.715
Random 24	0.587	0.344	−0.671	Random 49	0.401	0.161	−0.953
Random 25	0.754	0.569	−0.064	Random 50	0.721	0.519	−0.024
**Random Models Parameters**							
Average R	0.594						
Average R^2^	0.364						
Average Q^2^	−0.941						
cRp^2^	0.681						

**Table 4:** Docking results of the designed inhibitors and reference ligand with protein receptor.

Compound	Binding Affinity (kcal/mol)	Hydrogen-Binding Interaction	Hydrophobic Interaction	Electrostatic Interaction
**11a**	−8.18	ASP48. ASP181. SER216 [1.80–3.59]	ALA217. TYR46. CYS215. VAL49. [3.61–5.06]	ASP48 [3.89]
**11b**	−8.09	ASP181. TYR46. [2.88–2.98]	ALA217. TYR46. CYS215. LYS120. [3.12–4.88]	ASP48 [3.59]
**11c**	−8.30	ASP48, ASP181, SER216 [1.81–3.59]	ALA217. TYR46. CYS215. VAL49. ARG24.[3.49–5.25]	ASP48 [3.87]
**11d**	−7.97	ASP181. TYR20. SER216 [1.81–3.70]	ALA217. TYR46. PHE182. ILE219. [3.19–5.28]	ASP48 [3.48]
**11e**	−7.41	ASP181. TYR46. TYR20. [2.87–3.29]	ALA217. TYR46. CYS215. LYS120. PHE182. ILE219. [3.15–5.34]	ASP48 [3.47]
**11f**	−7.61	ASP48. SER216 [1.93–3.69]	ALA217. TYR46. CYS215. VAL49. PHE182. ARG221. [3.32–5.29]	ASP48 [3.85]
**11g**	−7.30	ASP48 [2.20–3.46]	ALA217. TYR46. CYS215. VAL49. PHE182. ILE219. ARG221. [3.64–5.39]	ASP48 [3.02]
**7e(ref)**	−7.19	ASP48. TYR46 [3.16–3.17]	ALA217. ARG221. CYS215. VAL49.	ASP48 [3.03]

**Table 5:** Physicochemical properties and bioavailability of the novel designed compounds and the reference ligand.

Compound	MW (g/mol)	Log P (Consensus)	Log S (ESOL)	GI Absorption	Bioavailability Score	Synthetic Accessibility	Lipinski	Pains
**11a**	455.32	2.60	−4.43	High	0.55	3.64	Yes	0
**11b**	469.35	2.94	−4.65	High	0.55	3.75	Yes	0
**11c**	441.30	2.27	−4.12	High	0.55	3.56	Yes	0
**11d**	471.32	1.97	−4.02	High	0.55	4.19	Yes	0
**11e**	471.32	2.11	−3.89	High	0.55	3.78	Yes	0
**11f**	458.37	3.63	−5.12	High	0.55	4.61	Yes	0
**11g**	456.35	3.55	−5.06	High	0.55	4.28	Yes	0
**7e(ref)**	440.35	5.247	−5.69	High	0.55	3.81	Yes	0

**Table 6:** Toxicity prediction results of the novel designed compounds.

Comp.	Hepatotoxicity	Carcinogenicity	Immunotoxicity	Mutagenicity	Cytotoxicity	Predicted LD_50(mg/kg)_	Class
**11a**	Inactive	Inactive	Inactive	Inactive	Inactive	1180	4
**11b**	Inactive	Inactive	Inactive	Inactive	Inactive	1180	4
**11c**	Inactive	Inactive	Inactive	Inactive	Inactive	1000	4
**11d**	Inactive	Inactive	Inactive	Inactive	Inactive	1190	4
**11e**	Inactive	Inactive	Inactive	Inactive	Inactive	1190	4
**11f**	Inactive	Inactive	Inactive	Inactive	Inactive	1400	4
**11g**	Inactive	Inactive	Inactive	Inactive	Inactive	1000	4

**Table 7:** All the results were obtained from VEGA QSAR of the novel designed compounds.

Compound	Mutagenicity (Ames Test)	Skin Irritation	Plasma Protein Binding	P-Glycoprotein Activity	Total Body Elimination Half-Life (Hour)
**11a**	No	No	0.806	Inactive	5.192
**11b**	No	No	0.939	Inactive	8.289
**11c**	No	No	0.596	Inactive	5.522
**11d**	No	No	0.557	Inactive	5.335
**11e**	No	No	0.503	Inactive	7.975
**11f**	No	No	1.125	Inactive	8.051
**11g**	No	No	1.070	Inactive	7.202
**7e(ref)**	No	No	1.427	Inactive	12.73

**Table 8:** Predicted biological activity of designed compounds using PASS webserver.

	Pharmacological Activity	Protein-Tyrosine Phosphatase Beta Inhibitor		Protein-Tyrosine Phosphatase 1B Inhibitor		Protein-Tyrosine Phosphatase Inhibitor		Antidiabetic		Antidiabetic Symptomatic		Antidiabetic (Type 2)	
Compounds		Pa	Pi	Pa	Pi	Pa	Pi	Pa	Pi	Pa	Pi	Pa	Pi
**11a**		0.343	0.002	0.173	0.016	0.391	0.005	0.377	0.050	0.333	0.025	0.130	0.109
**11b**		0.368	0.002	0.181	0.015	0.411	0.005	0.426	0.037	0.393	0.014	0.137	0.100
**11c**		0.356	0.002	0.180	0.015	0.400	0.005	0.382	0.048	0.336	0.024	0.132	0.107
**11d**		0.271	0.003	0.130	0.027	0.344	0.008	0.316	0.074	0.318	0.030	/	/
**11e**		0.368	0.002	0.180	0.015	0.420	0.005	0.414	0.040	0.421	0.011	/	/
**11f**		0.290	0.003	0.181	0.015	0.346	0.008	0.372	0.052	0.299	0.040	0.178	0.069
**11g**		0.326	0.003	0.214	0.011	0.381	0.006	0.456	0.031	0.330	0.026	0.215	0.053

**Table 9:** Frontier orbitals energies and reactivity descriptor values of the designed compounds.

Comp	E_HOMO_ (eV)	E_LUMO_ (eV)	Eg (eV)	ɳ (eV)	δ (eV^−1^)	μ (eV)	ω (eV)
**11a**	−6.028	−2.314	3.714	1.857	0.269	−4.171	4.683
**11b**	−8.339	−1.273	7.066	3.533	0.142	−4.806	3.268
**11c**	−6.276	−2.596	3.681	1.840	0.272	−4.436	5.347
**11d**	−6.265	−2.561	3.704	1.852	0.270	−4.413	5.257
**11e**	−6.346	−2.627	3.720	1.860	0.269	−4.487	5.412
**11f**	−6.001	−2.375	3.626	1.813	0.276	−4.188	4.836
**11g**	−5.971	−2.242	3.729	1.864	0.268	−4.107	4.523
**7e(ref)**	−6.149	−2.465	3.684	1.842	0.271	−4.307	5.034

**Table 10:** Structural features and PTB1B activity of 5-(substituted benzylidene) thiazolidine-2,4-dione derivatives.

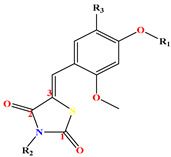				
Comp	R_1_	R_2_	R_3_	pIC_50_
**6a**	H	H	H	4.526
**6b**	H	H	Br	4.947
**6c**	CH_3_	H	Br	4.848
**6d**	–CH_2_–CH=CH_2_	H	Br	5.092
**6e**	–CH_2_–CH=C–(CH_3_)_2_	H	Br	5.276
**6f**	–CH_2_–(CH_2_)_2_–CH_3_	H	Br	5.310
**6g**	–CH_2_–C_6_H_5_	H	Br	5.119
**7a**	–CH_3_	CH_3_	Br	4.652
**7b**	–CH_2_–C_6_H_5_	CH_3_	Br	5.092
**7c**	–CH_2_–CH=C–(CH_3_)_2_	CH_3_	Br	5.086
**7d**	–CH_2_–CH=C–(CH_3_)_2_	–CH_2_–CH=CH_2_	Br	4.770
**7e(ref)**	–CH_2_–CH=C–(CH_3_)_2_	–CH–(CH_3_)_2_	Br	5.337
**7f**	–CH_2_–CH=C–(CH_3_)_2_	–CH_2_–(CH_2_)_2_–CH_3_	Br	4.910
**7g**	–CH_2_–CH=C–(CH_3_)_2_	–CH_2_–CH=C–(CH_3_)_2_	Br	5.000
**7h**	–CH_2_–CH=C–(CH_3_)_2_	–CH_2_–C_6_H_5_	Br	4.987
**7i**	–CH_2_–(CH_2_)_2_–CH_3_	CH_3_	Br	4.917
**7j**	–CH_2_–(CH_2_)_2_–CH_3_	–CH_2_–CH=CH_2_	Br	5.102
**7k**	–CH_2_–(CH_2_)_2_–CH_3_	–CH–(CH_3_)_2_	Br	5.013
**7m**	–CH_2_–(CH_2_)_2_–CH_3_	–CH_2_–C_6_H_5_	Br	5.180
**12**	–CH–O–(CH_2_)_4_ ^(*)^	H	–CH_2_–CH=CH_2_	4.914
**13d**	–CH–O–(CH_2_)_4_ ^(*)^	–CH_2_–CH=C–(CH_3_)_2_	–CH_2_–CH=CH_2_	4.644
**13e**	–CH–O–(CH_2_)_4_ ^(*)^	–CH_2_–(CH_2_)_2_–CH_3_	–CH_2_–CH=CH_2_	4.686
**14b**	H	–CH–(CH_3_)_2_	–CH_2_–CH=CH_2_	5.194
**14d**	H	–CH_2_–CH=C–(CH_3_)_2_	–CH_2_–CH=CH_2_	4.921
**14e**	H	–CH_2_–(CH_2_)_2_–CH_3_	–CH_2_–CH=CH_2_	5.018
**14f**	H	–CH_2_–C_6_H_5_	–CH_2_–CH=CH_2_	5.143
**14g**	H	H	–CH_2_–CH=CH_2_	4.533
*:	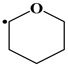			

**Table 11:** List of descriptors used in this work.

Descriptors	Symbol	Class
Molecular weight	MW	Constitutional
Coefficient of partition Octanol/Water	LogP	Physico-chemical
Solubility	LogS	
Polarizability	Pol	
Hydrogen Bond Acceptor	HBA	
Hydrogen Bond Donor	HBD	
Molar Refractivity	MR	Geometrical
Molar Volume	MV	
Energy Total	E_T_	Quantum (Electronic)
Energy HOMO	E_HOMO_	
Energy LUMO	E_LUMO_	
Charges	qn	Mulliken Charges
Charges	qS	
Charges	qC1	
Charges	qC2	
Charges	qC3	
Balaban Index	BIndx	Topological
Cluster Count	ClsC	
Molecular Topological Index	TIndx	

**Table 12:** Displays the molecular descriptors’ values that were employed in the regression analysis.

Comp	pIC_50_	LogP	MW	LogS	MR	MV	POL	E_HOMO_	E_LUMO_	E_T_	HBA	HBD	qS	qN	qC1	qC2	qC3	Clsc	Tindx	Bindx
**6a**	4.526	1.725	251.257	−3.55	68.34	679.733	24.44	−2.406	−6.114	−32,074.5	4	2	−0.371	−0.267	0.044	−0.116	0.643	17	3549	106,143
**6b**	4.947	2.487	330.153	−4.26	75.874	735.382	27.066	−2.585	−6.254	−102,106	4	2	−0.373	−0.26	0.046	−0.223	0.799	18	3884	136,497
**6c**	4.848	2.790	344.18	−4.38	80.643	782.485	28.901	−2.521	−6.137	−103,175	4	1	0.412	−0.262	0.083	−0.079	0.596	19	4627	177,387
**6d**	5.092	3.346	370.218	−5.04	89.806	877.383	32.379	−2.653	−6.337	−105,282	4	1	−0.03	−0.207	0.015	−0.228	0.62	21	6539	295,568
**6e**	5.276	4.127	398.271	−5.93	99.761	962.68	36.049	−2.604	−6.276	−107,422	4	1	−0.011	−0.207	0.008	−0.231	0.624	23	8921	472,150
**6f**	5.310	3.961	386.26	−5.67	94.516	947.696	34.406	−2.673	−6.319	−106,385	4	1	−0.17	−0.238	0.01	−0.18	0.589	22	7722	377,714
**6g**	5.119	4.361	420.277	−5.92	109.394	978.128	38.561	−2.607	−6.272	−109,464	4	1	−0.191	0.239	−0.016	−0.274	0.628	25	11,391	546,426
**7a**	4.652	3.132	358.207	−4.38	85.54	819.806	30.736	−2.428	−6.060	−104,245	4	0	0.166	0.032	0.082	−0.18	0.529	20	5375	224,427
**7b**	5.092	4.703	434.304	−5.94	114.291	1024.088	40.396	−2.512	−6.199	−110,534	4	0	−0.07	0.056	−0.047	−0.338	0.675	26	12,739	653,163
**7c**	5.086	4.469	412.298	−5.94	104.658	1030.626	37.884	−2.509	−6.201	−108,492	4	0	−0.07	0.056	−0.015	−0.295	0.542	24	10,061	573,023
**7d**	4.770	5.025	438.336	−5.94	113.821	1083.626	41.362	−2.486	−6.167	−110,599	4	0	−0.07	0.34	−0.034	−0.07	0.315	26	12,885	843,380
**7e(ref)**	5.337	5.247	440.352	−6.77	113.824	1107.71	41.554	−2.465	−6.149	−110,632	4	0	−0.231	0.412	−0.031	0.104	0.162	26	12,715	833,233
**7f**	4.910	5.639	454.379	−7.23	118.531	1151.608	43.389	−2.476	−6.166	−111,703	4	0	−0.073	0.391	−0.038	0.157	−0.092	27	14,584	1,020,188
**7g**	5.000	5.805	466.39	−7.49	123.776	1190.101	45.032	−2.443	−6.144	−112,739	4	0	−0.129	0.378	−0.021	0.032	0.149	28	16,299	1,217,684
**7h**	4.987	6.039	47.544	−7.49	133.409	1195.143	47.544	−2.504	−6.185	−114,781	4	0	−0.17	0.418	0.019	0.178	−0.107	30	19,801	1,300,406
**7i**	4.917	4.303	400.287	−5.68	99.413	999.466	36.241	−2.525	−6.227	−107,456	4	0	−0.067	0.057	0.002	−0.289	0.554	23	8758	462,362
**7j**	5.102	4.859	426.325	−6.35	108.576	1054.01	39.719	−2.515	−6.191	−109,562	4	0	−0.107	0.322	−0.012	−0.01	0.279	25	11,350	692,217
**7k**	5.013	5.081	428.341	−6.51	108.579	1077.561	39.911	−2.481	−6.174	−109,596	4	0	−0.235	0.411	−0.013	0.106	0.18	25	11,188	683,230
**7m**	5.180	5.873	476.385	−7.23	128.164	1165.816	45.901	−2.517	−6.033	−113,744	4	0	−0.201	0.383	0.029	0.281	−0.302	29	17,754	1,097,466
**12**	4.914	3.653	375.439	−5.86	104.67	1029.891	38.791	−2.272	−5.889	−42,615.8	5	1	−0.324	−0.253	0.036	−0.366	0.871	26	12,024	614,951
**13d**	4.644	5.332	443.558	−7.42	128.685	1274.358	47.774	−2.127	−5.772	−47,932.8	5	0	−0.167	0.364	−0.041	−0.069	0.435	31	20,797	1,445,876
**13e**	4.686	5.166	431.547	−7.16	123.44	1242.555	46.131	−2.154	−5.791	−46,896.2	5	0	−0.118	0.368	−0.046	0.104	0.183	30	18,773	1,230,606
**14b**	5.194	3.574	333.402	−5.49	95.931	938.266	35.258	−2.229	−5.880	−38,451.7	4	1	−0.348	0.369	0.027	0.227	0.096	23	8545	435,114
**14d**	4.921	4.132	359.44	−6.22	105.883	1015.594	38.736	−2.212	−5.880	−40,568.2	4	1	−0.209	0.338	−0.02	0.157	0.033	25	11,390	672,700
**14e**	5.018	3.966	347.429	−5.97	100.638	982.053	37.093	−2.237	−5.901	−39,531.6	4	1	−0.157	0.356	−0.041	0.293	−0.25	24	10,034	55,093
**14f**	5.143	4.366	381.446	−6.22	115.516	1025.745	41.248	−2.269	−5.927	−42,610.1	4	1	−0.22	0.399	0.019	0.301	−0.202	27	14,174	750,061
**14g**	4.533	2.453	291.321	−4.66	81.868	797.075	29.753	−2.363	−6.008	−35,251.2	4	2	−0.312	0.268	0.043	−0.166	0.521	20	5681	226,445

## Data Availability

Data are contained within the article.
